# Availability of Services and Caregiver Burden: Supporting Individuals With Neurogenetic Conditions During the COVID-19 Pandemic

**DOI:** 10.1177/08830738211001209

**Published:** 2021-04-08

**Authors:** Michelle Kowanda, Lindsey Cartner, Catherine Kentros, Alexa R. Geltzeiler, Kaitlyn E. Singer, W. Curtis Weaver, Christopher D. Lehman, Simone Smith, Rebecca Sheedy Smith, Lauren Kasparson Walsh, Katharine Diehl, Natalie Nagpal, Elizabeth Brooks, Caroline M. Mebane, Ashley L. Wilson, Alison R. Marvin, L. Casey White, J. Kiely Law, William Jensen, Amy M. Daniels, Jennifer Tjernagel, LeeAnne Green Snyder, Cora M. Taylor, Wendy K. Chung

**Affiliations:** 1 382904 Simons Foundation, New York, NY, USA; 2 Department of Pediatrics, 21611 Columbia University Irving Medical Center, New York, NY, USA; 3 2780 Geisinger Autism & Developmental Medicine Institute, Lewisburg, PA, USA; 4 Maryland Center for Developmental Disabilities, 27870 Kennedy Krieger Institute, Baltimore, MD, USA; 5 Department of Medicine, 21611 Columbia University Irving Medical Center, New York, NY, USA

**Keywords:** autism, neurogenetic, caregiver stress, telehealth, COVID-19 pandemic

## Abstract

Because of the COVID-19 pandemic, in-person services for individuals with neurodevelopmental disabilities were disrupted globally, resulting in a transition to remote delivery of services and therapies. For individuals with neurogenetic conditions, reliance on nonclinical caregivers to facilitate all therapies and care was unprecedented. The study aimed to (1) describe caregivers’ reported impact on their dependent’s services, therapies, medical needs, and impact on themselves as a result of the COVID-19 pandemic and (2) assess the relationship between the extent of disruption of services and the degree of self-reported caregiver burden. Two online questionnaires were completed by caregivers participating in Simons Searchlight in April and May 2020. Surveys were completed by caregivers of children or dependent adults with neurodevelopmental genetic conditions in Simons Searchlight. Caregivers reported that the impact of the COVID-19 pandemic moderately or severely disrupted services, therapies, or medical supports. The majority of caregivers were responsible for providing some aspect of therapy. Caregivers reported “feeling stressed but able to deal with problems as they arise,” and reported lower anxiety at follow-up. Caregivers reported that telehealth services were not meeting the needs of those with complex medical needs. Future surveys will assess if and how medical systems, educational programs, therapists, and caregivers adapt to the challenges arising during the COVID-19 pandemic.

After the declaration of the COVID-19 pandemic by the WHO on March 11, 2020, education, therapies, and medical care rapidly transitioned from in-person to remote worldwide. This rapid adjustment may be especially difficult for individuals with complex medical needs. Individuals with neurogenetic conditions and neurodevelopmental disorders often require intensive individualized early intervention^1,2^ and significant medical support.^3^ Children with neurogenetic conditions and neurodevelopmental disorders typically use more health care services than neurotypical children, with a larger number of prescriptions, physician visits, and subspecialists required for comprehensive care.^4,5^ Services in this population include medical, educational, and therapeutic intervention such as occupational, speech, or behavioral therapy. Similarly, adults with neurodevelopmental disorders have greater medical requirements. Furthermore, children with autism, commonly diagnosed in individuals with neurodevelopmental conditions, have a rigorous therapy schedule with a median of 6.0 hours/wk total for all therapies.^3^ Early studies of the impact of COVID-19 on children with autism indicated that children had more frequent or more intense behavior problems during the pandemic.^6^ Adults with autism are more likely to require physician visits and psychiatric support, have greater numbers of emergency psychiatric admissions compared to the general population,^7,8^ and report barriers to health care as well as unmet health care needs.^9^

Providing care for a child with a neurodevelopmental disorder may also impact the physical and psychological health of the caregiver,^10^ even under ordinary circumstances.^11^ Caregivers of children with neurodevelopmental disorders and behavioral issues are 2.1 times more likely to report a chronic health condition, and 3.67 times more likely to have somewhat/very elevated depressive symptoms,^12^ suggesting that caregiver’s mental and physical health is vulnerable to stressors and burdens. Additionally, caring for an individual with a neurodevelopmental disorder is a lifelong journey, and older caregivers who report a higher dependent developmental burden report a lower quality of life.^13^ Finally, the impact of the COVID-19 pandemic poses additional stressors to these caregivers of rare neurodevelopmental disorders who, even before the pandemic, reported experiencing social exclusion and isolation.^14^ These additional stressors may include a loss of in-home support staff, concern about a perceived increased COVID-19 complication risk for their dependent, and economic stressors from financial instability and resource insecurity.

The shift to remote service delivery during the COVID-19 pandemic forced families of individuals with neurodevelopmental disorder conditions to suddenly adjust to modifications of their usual support systems. This included switching to video or telephone consultations with general health and education practitioners, specialists, and occupational or speech therapists. Never before have children with neurodevelopmental disorders and their caregivers had to adapt to large-scale telemedicine implementation and reduced support from teachers and other professionals, all while balancing child care and employment and economic stressors, including working from home, furloughs, or losing a job. Prior literature suggests that the convenience of telemedicine and the potential for greater accessibility may improve patient-centered outcomes,^15^ with evidence that telemedicine-patient satisfaction has generally been high.^16^ However, these studies have not evaluated telemedicine utilization and satisfaction specifically in families with neurodevelopmental disorders. To address this, the Simons Searchlight study assessed the access to services and use of telemedicine in a large group of related neurodevelopmental disorders around the world with diverse medical needs during the beginning of the COVID-19 pandemic.

Our study surveyed caregivers of children and dependent adults with a genetic variant associated with genetic neurodevelopmental disorders. The purpose of the study was to describe caregiver-reported impact on various services for individuals previously receiving mostly in-person services prior to the COVID-19 pandemic. Additionally, the authors aimed to determine if caregivers reported their dependent benefiting from remote services and to assess the relationship between self-reported caregiver burden and the extent of disruption to services.

## Materials and Methods

### Participants

Simons Searchlight, formerly Simons Variation in Individuals Project (Simons VIP), is an international online research registry for individuals with a genetic diagnosis associated with autism and other neurodevelopmental disorders^17^ (see list of genetic conditions www.sfari.org/resource/simons-searchlight/). Caregivers enrolled in Simons Searchlight were invited by e-mail to complete a survey of the impact of the COVID-19 pandemic on services. Participants were eligible if they could read and understand English and had a living dependent with an active account enrolled in Simons Searchlight system. Only 1 survey was provided per caregiver; therefore, even if a caregiver had more than 1 dependent enrolled in Simons Searchlight, he or she was only asked about one dependent.

### Data Collection

Caregivers (n=1496) were invited to complete both a preliminary 37-item survey (survey I; April 1, 2020–April 14, 2020) and a 68-item follow-up survey (survey II; April 29, 2020–May 13, 2020; see Supplemental materials). Survey questions were developed by members of the Simons Searchlight research team. Survey I included questions on the impact of the COVID-19 pandemic on medical care, therapy, education, online delivery of services, and parent and dependent emotional and mental health. Survey I was also used as a guideline for refinement of questions in survey II.

Survey II repeated questions from survey I and added detailed questions about prepandemic services as well as use and effectiveness of online/remote services. Caregivers ranked to what extent their dependent was benefiting from each online or remote service, by selecting one of 4 options: significantly, moderately, minimally, or not at all. Adaptation of services was considered successful if 50% or more of caregivers ranked services received by the dependent as significantly or moderately benefiting their dependent.

Caregiver level of personal perceived emotional crisis was assessed in survey II using the Brief Family Distress Scale (BFDS). BFDS is one question where caregivers indicate their level of distress on a scale from 1 to 10. Crisis is interpreted within the published literature as none (scores 1-3), moderate (scores 4-5), or marked or near or in crisis (scores 6-10).^18^ Survey I and II also included core mental health questions from the Johns Hopkins Bloomberg School of Public Health Coronavirus and Mental Health Measurement Working Group. Caregivers’ “emotional or mental health” and caregivers’ feeling “nervous, anxious, or on edge” were collected through 2 different questions. Possible ranking of emotional or mental health was as follows: excellent, very good, good, fair, or poor. Caregiver feeling nervous, anxious, or on edge was ranked according to the following: rarely or none of the time (<1 day), some or a little of the time (1-2 days), occasionally or a moderate amount of time (3-4 days), or most or all of the time (5-7 days). Changes in overall caregiver response was compared between caregivers who completed both survey I and survey II.

Information relating to additional affected body systems were gathered from the Simons Searchlight database. Access to this database is available de-identified for researchers upon request through base.sfari.org. Most participants had previously completed a medical history interview and Vineland Adaptive Behavior Scales Parent Caregiver Interview Form Second Edition^19^ by phone interview as part of their participation in Simons Searchlight. The Vineland II Adaptive Behavior Composite (ABC) score ranges from 20 to 160 and consists of 4 domains: communication, daily living skills, socialization, and motor skills (for younger children).

### Analysis

The data were analyzed using Stata, version 12.1. The Vineland II ABC data were divided into 3 categories to stratify the group by the dependent’s functional level: >79, low average and above; 40-79, mildly to moderately impaired; and <40, severely to profoundly impaired. Descriptive statistics were used to characterize our cohort and describe services disrupted during the COVID-19 pandemic. Individuals who did not complete both survey I and survey II were not included in caregiver mental health assessments. McNemar’s test was applied to study the difference in caregiver stress over time, between survey I and survey II.

## Results

Of the 391 participants who completed the detailed 68-item follow up survey II, 301 caregivers completed both preliminary survey I and survey II. Participants who did not complete the description of the services section in survey II were removed from analysis. Respondents were mostly female (91.0%), living in the United States (72.9%) with dependents who were mostly male (59.1%) and 10 years old on average (SD of 6.5) (Table 1). Survey II respondents included individuals living in 28 different countries representing 60 different neurodevelopmental genetic conditions, with 16p11.2 deletion syndrome being the most common (18.2% of respondents) (Table S1 and S2). The distribution of individuals across countries represented in the survey data is comparable to the Simons Searchlight participants invited to take the survey. Dependents with complete medical history data were frequently reported to have seizures (34.8%), autism spectrum disorder (40.4%), and were moderately (77.3%) or severely (10.8%) impaired per the Vineland II (Table 1). Dependents were reported to have an average of between 5 and 6 body systems affected out of 16 considered and were taking an average of 3 medications (Table 1). The number of affected body systems and medications was an indicator of the level of medical complexity.

**Table 1.** Survey II Respondent and Dependent Demographic and Medical Characteristics (n = 391).

Respondents^a^	n (%) or mean (SD, range)
Caregiver sex, n (%)	
Male	35 (9.0)
Female	356 (91.0)
Country of residence, n (%)	
United States	285 (72.9)
Other	106 (27.1)
Dependent characteristics	
Age at survey, y^b^, n (%)	
≤5	117 (29.9)
6-17	240 (61.4)
≥18	34 (8.7)
Child sex, n (%)	
Male	231 (59.1)
Female	160 (40.9)
Reported genetic status, n (%)	
CNV	138 (35.3)
Monogenic condition	253 (64.7)
Reported seizures^c^, n (%)	
Yes	103 (34.8)
Reported autism spectrum disorder^d^, n (%)	
Yes	118 (40.4)
Vineland composite score,^e^ mean (SD), range	62.0 (17.2), 20-108
Vineland adaptive functioning, n (%)	
Severely to profoundly impaired	27 (10.8)
Mildly to moderately impaired	194 (77.2)
Average to above average	30 (12.0)
Comorbidity details, mean	
Body system score^f,g^	5.5
Current medications (range 0-14)^h^	2.9

^a^ The mean age of respondents was 42.0 (SD 7.6).

^b^ The mean age of the dependents (children) was 10.1 (SD 6.5)

^c^ For this variable, n = 296

^d^ For this variable, n = 292

^e^ For this variable, n *=* 251

^f^ For this variable, n = 295, SD of 2.2

^g^ Systems considered for the score include allergy, immunology, orthopedic, cancer, dermatologic, endocrinologic, gastrointestinal, genital, cardiac, infectious diseases, renal, neurologic, pulmonary, seizures, and ophthalmologic.

^h^ For this variable, n = 262.

**Figure 1.**
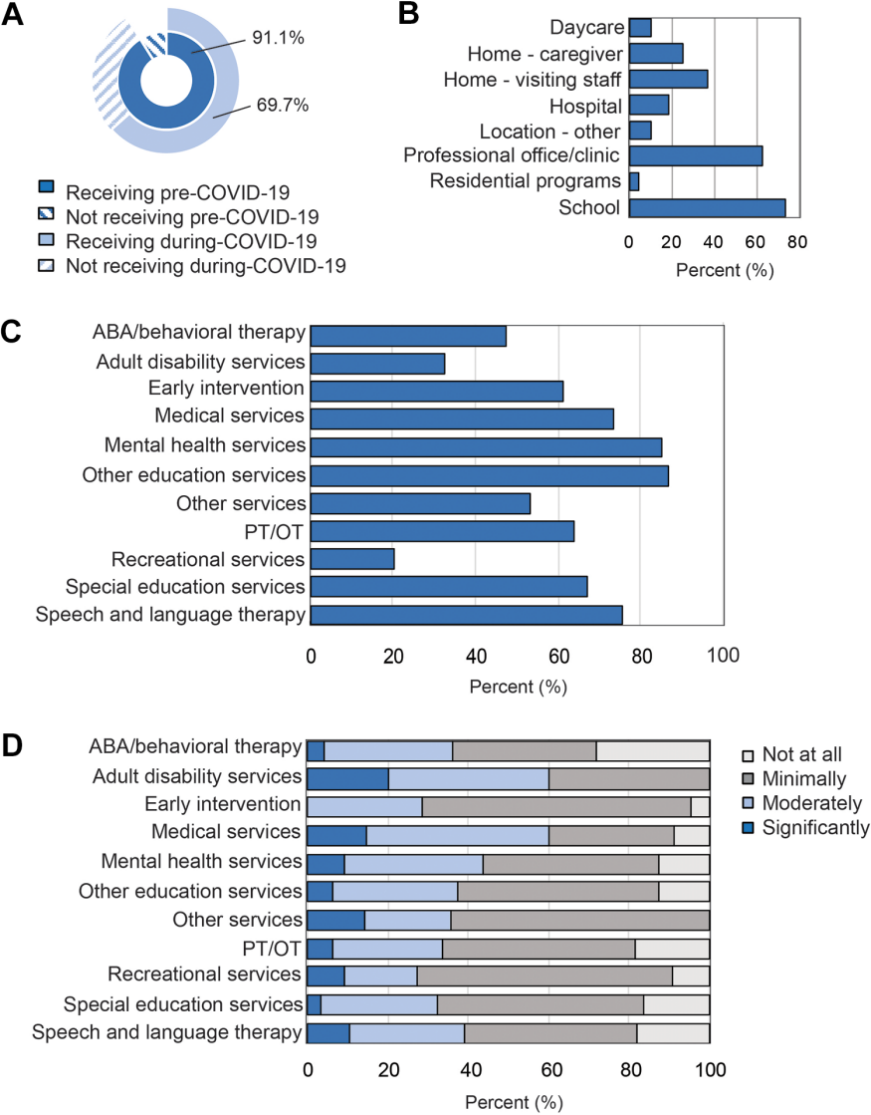
(A) Receipt of in-person services pre-COVID-19 and subsequently provided through online/remote systems during the COVID-19 pandemic. (B) Locations of pre-COVID-19 services. (C) Percentage of individuals receiving therapies, services, and medical supports remotely during COVID-19. (D) Caregivers rated the benefit of receiving various services remotely.

Prior to shutdowns related to the COVID-19 pandemic, most dependents were reported to be receiving services, therapies, or medical support in-person (91.0%, Figure 1A). At that time, some caregivers reported administering therapies for their dependent at home (24.7%) (Figure 1B). Additionally, most services were received in school (73.0%) and clinical settings (62.7%) (Figure 1B). Of those who were receiving services, many caregivers reported receiving services in a remote or online setting during the COVID-19 pandemic (69.7%, Figure 1A). At the time of survey II, caregivers reported a decrease in access to all services (Figure 1C).

Most caregivers reported that their dependent’s services, therapies, or medical care were moderately to severely disrupted (87.1%), with an average of 47.2 days since service disruption by the time of survey II completion in May 2020. Caregivers whose dependents were receiving services remotely perceived low benefit of remote services, most ranking services as minimally or not at all effective (Figure 1D). Additionally, most caregivers reported they were responsible for providing at least some aspect of therapy (73.2%); 36.7% of these services were rated as successfully adapted or modified. In general, most caregivers did not find online therapies beneficial for their dependent, with the exception of specific medical services (60.3%, n = 68) and adult disability services (60.0%, n = 5) (Figure 1D). General practitioners were accessed more than other physicians (29.7%), and most physician subspecialists provided their services through telehealth platforms (Table S3). Additionally, caregivers were more likely to postpone appointments than procedures (Table S4). Caregivers did not report disruption in access to medications or communication with physicians.

**Figure 2.**
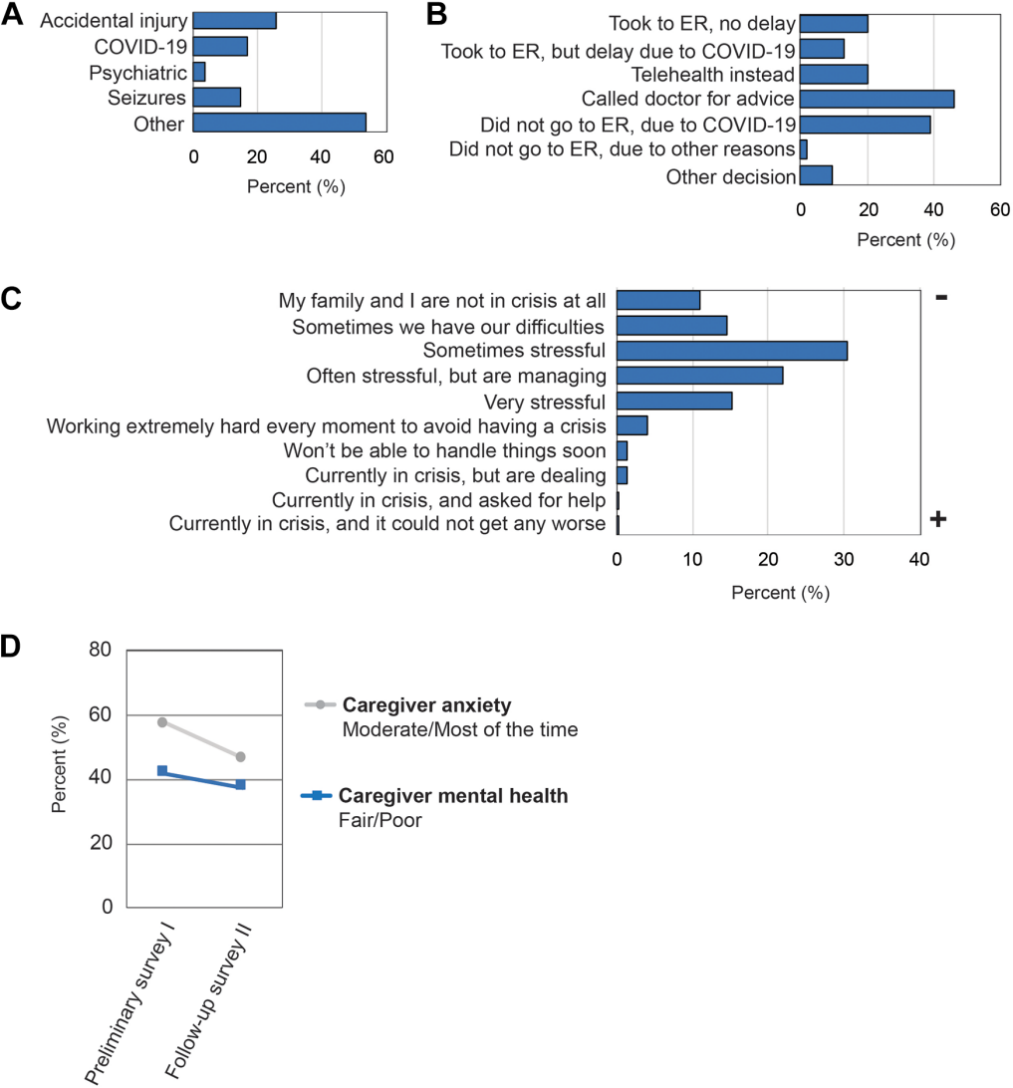
(A) Percentage of individuals considered for emergency department visits during COVID-19 by indication. (B) Action taken for the medical concern for which emergency department visit was considered. (C) Caregiver-reported adjustment to COVID-19 impact at survey II. (D) Percentage of caregivers with anxiety and fair or poor mental health.

The use of emergency services for medically complicated individuals was explored; the most common reason for caregivers to consider taking their dependent to the emergency department (n = 54) was accidental injury (25.9%), suspected COVID-19 infection (16.7%), seizures (14.8%), or a reason other than COVID-19 infection (53.7%) (Figure 2A). Of the caregivers who considered going to the emergency department, 32.7% ended up going to the emergency department. Only 22.2% of those who ended up going to the emergency department called the doctor prior (22.2% of the 32.7%). Of the caregivers who considered the need for an emergency department visit, 38.9% decided not to go because of fear of COVID-19, and 46.3% of caregivers responded that they were able to call a medical doctor for support (Figure 2b). Few caregivers responded that someone in their household had symptoms that were concerning for COVID-19, that is, 9.1% and 2.5% in survey I and II, respectively. Additionally, 1.8% of caregivers at survey I and 1.3% of caregivers at survey II responded that someone in the household tested positive for a COVID-19 infection.

Self-reported caregiver burden and its association with service disruption were queried. About half (46.5%) of caregivers who responded to survey II were working remotely: 41.9% reported that the impact of the COVID-19 pandemic had a negative impact on their employment and 33.3% experienced a subsequent negative impact on finances. Some caregivers (33.1%) reported that they were minimally or not at all coping with the additional responsibility of providing education, services, or therapies. Additionally, 77.8% of caregivers were either extremely or moderately overwhelmed by the disruptions in their child’s services. However, when ranking the current overall crisis level, most caregivers reported a 3 out of a maximum of 10; “Things are sometimes stressful, but we can deal with problems if they arise” (Figure 2C). To observe the change in caregiver stress during the COVID-19 pandemic, caregivers were provided core mental health questions in both survey I and survey II. A total of 301 caregivers completed mental health questions in both sets of surveys. Although caregivers’ “emotional or mental health” did not significantly change over time (survey I: 41.9%, survey II: 37.6%, χ^2^ (1, n = 301) = 2.3, *P* = .13), caregivers feeling “nervous, anxious, or on edge” improved significantly (survey I: 57.2%, survey II: 46.5%, χ^2^(1, n = 301) = 12.2, *P* < .001; Figure 2D).

## Discussion

Genetic neurodevelopmental disorder conditions are heterogeneous and associated with intellectual, developmental, and learning disabilities, or autism spectrum disorder, and supports were disrupted by the COVID-19 pandemic and shelter in place orders. During the COVID-19 pandemic, many individuals with genetic neurodevelopmental disorders receiving medical services and support from a team of specialists lost access to in-person resources. Our survey was not specific about the level of disruption; therefore, disruption may indicate a loss of services or a decrease in regular services. In general, in-home therapies are more common when children with disabilities are below the age of 3, but such therapies are less common for older children with disabilities, who receive therapies either in an outpatient setting or in school.^20^ Prepandemic, caregivers reported services were most often provided at schools and professional clinics, with social distancing and shelter in place orders pausing services at these locations. Furthermore, a low percentage of caregivers reported administering services themselves at home prepandemic, suggesting that caregivers were tasked to develop new skills to continue care for dependents receiving services remotely. A concern of the continued lack of in-person professional supportive services is that dependents may experience a loss or plateau of skills previously acquired. The long-term effects on skill development is unknown, especially for younger children, nor is it known how long it will take for dependents to regain their skills if they are lost due to disruptions to service. Future studies will be required to monitor outcomes long term. Furthermore, as support services disruption may continue in many areas due to the impact of the COVID-19 pandemic, it is likely that caregivers will continue to be required to facilitate therapies and services. Finally, therapists may consider providing parents with additional training sessions in order to ensure adequate support for their dependents.

Given the urgency of social distancing, the establishment of remote services, therapies and medical supports may contribute to caregiver stress and burden, as shown in our findings. Caregiver stress and ability to adapt are shaped by a combination of factors including child characteristics, psychological characteristics of the caregiver, socioeconomic status and social support.^21^ Surveys of caregivers of individuals with a genetic diagnosis or a neurodevelopmental diagnosis found that dependents lost a substantial number of educational and medical supports during the COVID-19 pandemic.^22^ This was suggested to add to caregiver burden in addition to negatively impacting both the caregiver’s and dependent’s overall health and well-being.^22^ Families with children who have physical disabilities, such as cerebral palsy (42%) or neuromuscular diseases (11%) reported that for more than 60% of children, parents performed the therapy,^23^ compared to 73.2% of parents providing therapies in our study. Also, caregivers for dependents with cerebral palsy report mental load was the most common daily parental difficulty (50% of parents reporting this issue).^23^ Caregivers of persons with Alzheimer’s disease found that the confinement as a result of the COVID-19 pandemic resulted in increased caregiver stress, and notably most family members discontinued cognitive and physical therapies.^24^ Finally, surveyed caregivers of adults with intellectual disability had significantly greater levels of a wish fulfilment coping style, defeat/entrapment, anxiety and depression.^25^

In general, children are resilient to SARS-CoV-2 infection.^26,27^ Adults and household members with comorbidities are at greater risk of complications with COVID-19.^28^ It has been posited that children with neurodevelopmental disorders are at increased risk, for several reasons including prevalence of comorbid conditions associated with neurodevelopmental disorders, the reliance on caregivers for care and potential difficulty adherence to public health measures. Early studies have found children with neurodevelopmental disorder conditions are among those hospitalized children with acute COVID-19.^29^ Finally, dependents with intellectual and developmental disability between the ages of 0 and 17 years were found to be at increased risk of COVI-19 infection.^30^ As in-person services become available in some areas of the world in the coming months, medical staff, therapists and other service supports should utilize face masks and other forms of personal protective equipment shown to effectively limit transmission.^31^ With appropriate precautions, symptom surveillance, testing for SARS-CoV-2, and isolation of infected individuals, the benefit of some in-person therapies for some children may outweigh the risks of long-term social isolation at critical times in development.

A limitation of this study is that participation was restricted to English speakers only, excluding individuals who do not speak English within the United States and around the world from participating in the study. Furthermore, to participate in Simons Searchlight, participants require a genetic diagnosis and access to genetic testing to get a diagnosis. Finally, this is a population of individuals interested in participating in research. Also, small sample sizes for most genetic conditions made it difficult to identify patterns for individual genetic subgroups.

## Conclusion

Caregivers reported a disruption in services, therapies, or medical supports due to the COVID-19 pandemic. Furthermore, remote services, although the only option at the time, were not perceived beneficial for dependents by most caregivers. The disruption to dependent’s services left most caregivers either extremely or moderately overwhelmed. As the impact of the loss of in-person services is unknown, follow-up investigation after the resolution of the pandemic will be valuable. We plan to continue to follow participants in the Simons Searchlight community longitudinally for at least the next year to determine the impact of this pandemic on this group of related genetic neurodevelopmental disorders.

## Supplemental Material

Supplemental Material, sj-pdf-1-jcn-10.1177_08830738211001209 - Availability of Services and Caregiver Burden: Supporting Individuals With Neurogenetic Conditions During the COVID-19 Pandemic Click here for additional data file. Supplemental Material, sj-pdf-1-jcn-10.1177_08830738211001209 for Availability of Services and Caregiver Burden: Supporting Individuals With Neurogenetic Conditions During the COVID-19 Pandemic by Michelle Kowanda, Lindsey Cartner, Catherine Kentros, Alexa R. Geltzeiler, Kaitlyn E. Singer, W. Curtis Weaver, Christopher D. Lehman, Simone Smith, Rebecca Sheedy Smith, Lauren Kasparson Walsh, Katharine Diehl, Natalie Nagpal, Elizabeth Brooks, Caroline M. Mebane, Ashley L. Wilson, Alison R. Marvin, L. Casey White, J. Kiely Law, William Jensen, Amy M. Daniels, Jennifer Tjernagel, LeeAnne Green Snyder, Cora M. Taylor and Wendy K. Chung in Journal of Child Neurology

Supplemental Material, sj-pdf-2-jcn-10.1177_08830738211001209 - Availability of Services and Caregiver Burden: Supporting Individuals With Neurogenetic Conditions During the COVID-19 Pandemic Click here for additional data file. Supplemental Material, sj-pdf-2-jcn-10.1177_08830738211001209 for Availability of Services and Caregiver Burden: Supporting Individuals With Neurogenetic Conditions During the COVID-19 Pandemic by Michelle Kowanda, Lindsey Cartner, Catherine Kentros, Alexa R. Geltzeiler, Kaitlyn E. Singer, W. Curtis Weaver, Christopher D. Lehman, Simone Smith, Rebecca Sheedy Smith, Lauren Kasparson Walsh, Katharine Diehl, Natalie Nagpal, Elizabeth Brooks, Caroline M. Mebane, Ashley L. Wilson, Alison R. Marvin, L. Casey White, J. Kiely Law, William Jensen, Amy M. Daniels, Jennifer Tjernagel, LeeAnne Green Snyder, Cora M. Taylor and Wendy K. Chung in Journal of Child Neurology

Supplemental Material, sj-pdf-3-jcn-10.1177_08830738211001209 - Availability of Services and Caregiver Burden: Supporting Individuals With Neurogenetic Conditions During the COVID-19 Pandemic Click here for additional data file. Supplemental Material, sj-pdf-3-jcn-10.1177_08830738211001209 for Availability of Services and Caregiver Burden: Supporting Individuals With Neurogenetic Conditions During the COVID-19 Pandemic by Michelle Kowanda, Lindsey Cartner, Catherine Kentros, Alexa R. Geltzeiler, Kaitlyn E. Singer, W. Curtis Weaver, Christopher D. Lehman, Simone Smith, Rebecca Sheedy Smith, Lauren Kasparson Walsh, Katharine Diehl, Natalie Nagpal, Elizabeth Brooks, Caroline M. Mebane, Ashley L. Wilson, Alison R. Marvin, L. Casey White, J. Kiely Law, William Jensen, Amy M. Daniels, Jennifer Tjernagel, LeeAnne Green Snyder, Cora M. Taylor and Wendy K. Chung in Journal of Child Neurology
