# Supplementary material for: Availability of Services and Caregiver Burden: Supporting Individuals With Neurogenetic Conditions During the COVID-19 Pandemic
Source: J Child Neurol. 2021 Apr 8;36(9):760–7. doi: 10.1177/08830738211001209 (PMC8033176; doi:10.1177/08830738211001209)
Supplement: Supplemental Material, sj-pdf-1-jcn-10.1177_08830738211001209 - Availability of Services and Caregiver Burden: Supporting Individuals With Neurogenetic Conditions During the COVID-19 Pandemic [file sj-pdf-1-jcn-10.1177_08830738211001209.pdf]

Supplemental Materials

Supplemental Table 1. Respondent country during COVID-19 survey II (May 2020, *n* = 391)

Country	*n (%)*
**United States**	285 (72.9%)
**United Kingdom**	31 (7.9%)
**Canada**	17 (4.4%)
**Australia**	14 (3.6%)
**Germany**	8 (2.1%)
**France**	4 (1.0%)
**Ireland**	4 (1.0%)
**Netherlands**	3 (0.8%)
**Spain**	3 (0.8%)
**Israel**	2 (0.5%)
**Sweden**	2 (0.5%)
**Austria**	1 (0.3%)
**Brazil**	1 (0.3%)
**Bulgaria**	1 (0.3%)
**Cyprus**	1 (0.3%)
**Greece**	1 (0.3%)
**India**	1 (0.3%)
**Italy**	1 (0.3%)
**Macao**	1 (0.3%)
**Mexico**	1 (0.3%)
**New Zealand**	1 (0.3%)
**Norway**	1 (0.3%)
**Poland**	1 (0.3%)
**Portugal**	1 (0.3%)
**Puerto Rico**	1 (0.3%)
**Russian Federation**	1 (0.3%)
**South Africa**	1 (0.3%)
**Suriname**	1 (0.3%)
**Switzerland**	1 (0.3%)

Supplemental Table 2. Dependent's genetic status (*n* = 319)

Genetic Status	*n (%)*
**16p11.2 deletion**	71 (18.2%)
**SCN2A**	30 (7.7%)
**16p11.2 duplication**	24 (6.1%)
**GRIN2B**	21 (5.4%)
**ASXL3 (Bainbridge-Ropers)**	20 (5.1%)
**PPP2R5D**	20 (5.1%)
**STXBP1**	16 (4.1%)
**1q21.1 deletion**	13 (3.3%)
**SYNGAP1**	13 (3.3%)
**CSNK2A1 (Okur-Chung)**	12 (3.1%)
**7q11.23 duplication**	9 (2.3%)
**DYRK1A**	9 (2.3%)
**1q21.1 duplication**	8 (2.1%)
**CHAMP1**	8 (2.1%)
**MED13L**	8 (2.1%)
**SETBP1 (Schinzel-Giedion)**	8 (2.1%)
**SLC6A1**	8 (2.1%)
**CTNNB1**	7 (1.8%)
**HIVEP2**	7 (1.8%)
**PACS1 (Schuurs-Hoeijmakers)**	7 (1.8%)
**16p11.2 deletion DISTAL**	6 (1.5%)
**ADNP (Helsmoortel-van der Aa)**	6 (1.5%)
**HNRNPH2**	5 (1.3%)
**TRIP12**	4 (1.0%)
**16p11.2 duplication DISTAL**	3 (0.8%)
**ARID1B (Coffin-Siris)**	3 (0.8%)
**CHD2**	3 (0.8%)
**PPP2R1A**	3 (0.8%)
**17q12 duplication**	2 (0.5%)

**ASH1L**	2 (0.5%)
**GRIN1**	2 (0.5%)
**GRIN2A**	2 (0.5%)
**KCNQ2**	2 (0.5%)
**KDM5B**	2 (0.5%)
**SETD5**	2 (0.5%)
**15q11.2 BP1-BP2 deletion**	1 (0.3%)
**3q29 deletion**	1 (0.3%)
**ANKRD11 (KBG)**	1 (0.3%)
**AUTS2**	1 (0.3%)
**BCL11A**	1 (0.3%)
**CASZ1**	1 (0.3%)
**CHD3**	1 (0.3%)
**CHD8**	1 (0.3%)
**CTBP1**	1 (0.3%)
**DNMT3A (Tatton-Brown-Rahman)**	1 (0.3%)
**FOXP1**	1 (0.3%)
**GIGYF1**	1 (0.3%)
**HNRNPU**	1 (0.3%)
**KMT2E (O'Donnell-Luria-Rodan)**	1 (0.3%)
**MBD5**	1 (0.3%)
**NLGN4x**	1 (0.3%)
**NRXN1**	1 (0.3%)
**POGZ (White-Sutton)**	1 (0.3%)
**PPP2R5C**	1 (0.3%)
**PTCHD1**	1 (0.3%)
**PURA**	1 (0.3%)
**SHANK3**	1 (0.3%)
**TAOK1**	1 (0.3%)
**USP9X**	1 (0.3%)
**VPS13B**	1 (0.3%)

Supplemental Table 3. During COVID-19 some dependents required access to physicians and caregivers and responded if they received telehealth services, in-person at home services, in-person at the physician office or if they could not access these medical services (*n* = 319).

**Physician**	**Needed to access *n (%)***	**Accessed through - *n (%)***			
		Telehealth	At-home	In-person office	Could not access
**General practitioner**	116 (29.7)	67 (57.8)	1 (0.9)	42 (36.2)	6 (5.2)
**Psychiatry**	28 (7.2)	23 (82.1)	1 (3.6)	2 (7.1)	2 (7.1)
**Neurology**	66 (16.9)	52 (80)	0	5 (7.7)	8 (12.3)
**Gastroenterology**	33 (8.4)	20 (60.6)	0	2 (6.1)	11 (33.3)
**Psychology**	22 (5.6)	11 (50)	1 (4.6)	5 (22.7)	5 (22.7)
**Other specialty**	85 (21.7)	33 (38.8)	3 (3.5)	17 (20)	32 (37.7)

Supplemental Table 4. During COVID-19 caregivers reported if they postponed appointments or procedures for their dependents (*n* = 319).

	**Yes *n (%)***	**No *n (%)***
**Appointments postponed**	255 (65.22)	136 (34.8)
**Procedures postponed**	134 (34.3)	257 (65.7)
