# Supplementary material for: Availability of Services and Caregiver Burden: Supporting Individuals With Neurogenetic Conditions During the COVID-19 Pandemic
Source: J Child Neurol. 2021 Apr 8;36(9):760–7. doi: 10.1177/08830738211001209 (PMC8033176; doi:10.1177/08830738211001209)
Supplement: Supplemental Material, sj-pdf-2-jcn-10.1177_08830738211001209 - Availability of Services and Caregiver Burden: Supporting Individuals With Neurogenetic Conditions During the COVID-19 Pandemic [file sj-pdf-2-jcn-10.1177_08830738211001209.pdf]

SIMONS SEARCHLIGHT

Impact of Coronavirus/COVID-19 on Services and Mental Health

The following survey includes questions about the impact of the novel coronavirus/COVID-19 on your child's (or dependent's) medical and therapeutic services and mental health. There are also questions about your mental health and your family's experiences as related to COVID-19 policies and news.

For families who have more than one child with the genetic change registered in Simons Searchlight, we have randomly selected ONE child as the participant in this study. We are unable to change the selected child. **Please answer the survey questions with the selected child/dependent in mind.**

Section 1: About your child/dependent's services

In the past week, to what extent have ChildFN's services, therapies or medical supports been disrupted due to COVID-19?

- ○ Severely
- ○ Moderately
- ○ Minimally
- ○ Not at all
- ○ Not applicable; My child doesn't receive services, therapies or medical supports

How many days have passed since ChildFN's services, therapies or medical supports were rst disrupted?

[ ] calendar days

Which of the following *settings* for services, therapies or medical support have been disrupted? *Select all that apply.*

- ☐ School
- ☐ Professional clinic or o ce
- ☐ Hospital
- ☐ Daycare
- ☐ Residential programs
- ☐ Home (administered by visiting sta)
- ☐ Home (administered by parent or caregiver)
- Other

Please specify "other" settings:

Which of the following best describes the current status of ChildFN's school (includes all school levels)? *If not in school, select "not applicable".*

- ○ Closed due to COVID-19
- ○ Closed due to previously scheduled spring break or other reason
- ○ Open
- ○ Not applicable; not in school

Which of the following *types* of services, therapies or medical supports have been disrupted? *Select all that apply.*

- ☐ Early intervention services
- ☐ ABA services or other behavioral therapy
- ☐ Mental health services
- Medical services
- ☐ Speech and Language therapy
- ☐ Physical or Occupational therapy
- ☐ Special education services
- ☐ Other education services
- ☐ Recreational services
- ☐ Adult disability services
- ☐ Special transportation services
- Other

Do you have concerns about ChildFN's physical health as a result of recent medical service disruptions?

Please specify "other" types of services, therapies or medical supports:

Overall, what percentage of ChildFN's services, therapies or medical supports have been disrupted due to COVID-19?

0 10 20 30 40 50 60 70 80 90 100

Overall, what percentage of ChildFN's services, therapies or medical supports have been successfully adapted or modi ed in response to the current disruptions?

0 10 20 30 40 50 60 70 80 90 100

To what extent have disruptions in services, therapies or medical supports negatively impacted ChildFN's symptoms, behaviors or other challenges?

- Severely
- Moderately
- Minimally
- Not at all

To what extent do you feel stressed or overwhelmed by the disruption in ChildFN's services, therapies or medical supports?

- Extremely
- Moderately
- Minimally
- Not at all

Currently, many school systems and professionals are implementing online or remote delivery of services, therapies and medical supports. Is ChildFN currently receiving any services, therapies or medical supports using this approach?

- Yes
- No

Has ChildFN speciFcally had a telemedicine visit since the disruption of services began? This would be an appointment with a healthcare professional over phone or video, that typically would occur in the provider oce or hospital.

- Yes
- No

To what extent do you think ChildFN is benetting from services, therapies or medical supports delivered online or remotely?

- SigniIcantly
- Moderately
- Minimally
- Not at all

As a result of the disruption in services, therapies or medical supports, have you or ChildFN's other parent/guardian had to take a full or partial leave from work to take care of ChildFN at home?

- Yes
- No

As a result of the disruption in services, therapies or medical supports for ChildFN, have you brought in other individuals to help in the home (relatives, home nurse, etc)?

- Yes
- No

To what extent do you think ChildFN is benetting from the support from additional individuals in the home?

- SigniIcantly
- Moderately
- Minimally
- Not at all

We are interested in hearing your ideas. Do you have any suggestions for ways that medical or service professionals could help meet your child/dependent's service or therapy needs during this time?

NEXT

You may close this window at any time to **Save and Exit**.

Any data entered on this page will be lost. All previous pages will be saved.

SIMONS SEARCHLIGHT

Impact of Coronavirus/COVID-19 on Services and Mental Health

Section 2: About your emotional or mental health

The following set of questions are asking about YOU and how well YOU are handling the current circumstances.

During the past week, how would you describe your own emotional or mental health?

- ◯ Excellent
- ◯ Very good
- ◯ Good
- ◯ Fair
- ◯ Poor

To what extent has your own emotional or mental health been negatively impacted by COVID-19?

- ◯ Severely
- ◯ Moderately
- ◯ Minimally
- ◯ Not at all

<u>In the past week</u>, how often have you felt nervous, anxious, or on edge?

- ◯ Rarely or none of the time (less than 1 day)
- ◯ Some or a little of the time (1-2 days)
- ◯ Occasionally or a moderate amount of time (3-4 days)
- ◯ Most or all of the time (5-7 days)

In the past week, how often have you felt depressed?

- Rarely or none of the time (less than 1 day)
- Some or a little of the time (1-2 days)
- Occasionally or a moderate amount of time (3-4 days)
- Most or all of the time (5-7 days)

In the past week, how often have you felt lonely?

- Rarely or none of the time (less than 1 day)
- Some or a little of the time (1-2 days)
- Occasionally or a moderate amount of time (3-4 days)
- Most or all of the time (5-7 days)

In the past week, how often have you felt hopeful about the future?

- Rarely or none of the time (less than 1 day)
- Some or a little of the time (1-2 days)
- Occasionally or a moderate amount of time (3-4 days)
- Most or all of the time (5-7 days)

In the past week, how often have you had physical reactions, such as sweating, trouble breathing, nausea, or a pounding heart, when thinking about your experience (e.g., social distancing, loss of income/work, concerns about infection) with the coronavirus/COVID-19 pandemic?

- Rarely or none of the time (less than 1 day)
- Some or a little of the time (1-2 days)
- Occasionally or a moderate amount of time (3-4 days)
- Most or all of the time (5-7 days)

Has a doctor or other healthcare provider ever told you that you have a mental health condition?

- Yes
- No

PREVIOUS | NEXT

You may close this window at any time to **Save and Exit**.

Any data entered on this page will be lost. All previous pages will be saved.

SIMONS SEARCHLIGHT

Impact of Coronavirus/COVID-19 on Services and Mental Health

Section 3: Other information

What is your ZIP/Postal Code?

____

Which of these recommendations did you follow during the past week? ***Select all that apply.***

- ☐ Avoid crowded places
- ☐ Avoid public places
- ☐ Keep your distance from others (6 feet)
- ☐ Change school or work arrangements
- ☐ Quarantine yourself if you have symptoms
- ☐ None

In the past week, has anyone in your household had symptoms that were concerning for COVID-19?

- ○ Yes
- ○ No

Has anyone in your family or household tested positive for COVID-19?

- ○ Yes
- ○ No

Do you know anyone personally (outside of your family or household) who has tested positive for COVID-19?

- Yes
- No

Do you know anyone personally who has been hospitalized from COVID-19?

- Yes
- No

Do you know anyone personally who has died from COVID-19?

- Yes
- No

Overall, how concerned are you about the impact of COVID-19 on your family or household?

- Extremely
- Moderately
- Minimally
- Not at all

Is there any additional information you would like to share about the impact of COVID-19 on you, your child or your family? ***For instance, what are you currently doing, or thinking about doing, to cope with or adapt to the changes imposed on you and your family due to COVID-19?***

Do you have any successful strategies that you would like to share? What's working for you and your family?

PREVIOUS SUBMIT

You may close this window at any time to **Save and Exit**.

Any data entered on this page will be lost. All previous pages will be saved.
