# Supplementary material for: Availability of Services and Caregiver Burden: Supporting Individuals With Neurogenetic Conditions During the COVID-19 Pandemic
Source: J Child Neurol. 2021 Apr 8;36(9):760–7. doi: 10.1177/08830738211001209 (PMC8033176; doi:10.1177/08830738211001209)
Supplement: Supplemental Material, sj-pdf-3-jcn-10.1177_08830738211001209 - Availability of Services and Caregiver Burden: Supporting Individuals With Neurogenetic Conditions During the COVID-19 Pandemic [file sj-pdf-3-jcn-10.1177_08830738211001209.pdf]

SIMONS SEARCHLIGHT

Impact of COVID-19 on Services and Mental Health (Follow-up 1)

This survey is the second in a series of Simons Searchlight surveys to look at the impact of the novel coronavirus (COVID-19) on your child’s (or dependent’s) medical and therapeutic services and mental health. We are interested in Vnding out about what online and remote therapies are working, or not working, for your family. We hope these answers will help families improve their own experience and provide input we can share with advocacy groups and service providers. There are also questions about your mental health and about COVID-19 symptoms and testing.

You may skip any question or stop answering these surveys at any time. Please note that no mental health professional will review any individual survey responses. If you are having serious emotional health diQculties, we strongly encourage you to (1) tell someone in your life about your feelings and concerns, and (2) to follow up with a national mental health resource such as the National Suicide Prevention Lifeline at 1-800-273-8255 (https://suicidepreventionlifeline.org/chat/ (https://suicidepreventionlifeline.org/chat/)) for US participants, or Befrienders.org (https://www.befrienders.org/) (to Vnd the resource number in your country).

For families who have more than one child with the genetic change registered in Simons Searchlight, we have selected only one of your children for this survey. We are unable to change the selected child. **Please answer the survey questions with this child/dependent in mind.**

Section 1: Impact on your child/dependent's services, therapies or medical supports

In the questions below, the phrase "services, therapies or medical supports" refers broadly to all services or therapies (e.g., medical, mental health, educational, and other supports) that your child/dependent might receive due to his/her diagnosis.

Thinking back to January/February 2020 (before COVID-19 changes), was ChildFN receiving any services, therapies or medical supports?

- Yes
- No

Thinking back to January/February 2020 (before COVID-19 changes), which of the following *types* of <u>services, therapies or medical supports</u> was ChildFN regularly receiving? *Check all that apply.*

- Early intervention services
- ABA services or other behavioral therapy
- Mental health services
- Medical services
- Speech and Language therapy
- Physical or Occupational therapy
- Special education services
- Other education services
- Recreational services
- Adult disability services
- Special transportation services
- Other

Thinking back to January/February 2020 (before COVID-19 changes), in which of the following <u>settings</u> did ChildFN receive services, therapies or medical supports? *Check all that apply.*

- School
- Professional clinic or o ce
- Hospital
- Daycare
- Residential programs
- Home (administered by visiting sta)
- Home (administered by parent or caregiver)
- Other

In the past week, to what extent have ChildFN's services, therapies or medical supports been disrupted due to COVID-19?

- Severely
- Moderately

- ◯ Minimally
- ◯ Not at all

How many days have passed since ChildFN's services, therapies or medical supports were Grst disrupted?

[ ] calendar days

Currently, what percentage of ChildFN's services, therapies or medical supports are disrupted due to COVID-19?

Click on the slider bar below to record your response. The color will change from gray to blue.

0 10 20 30 40 50 60 70 80 90 100

Currently, what percentage of ChildFN's services, therapies or medical supports have been successfully adapted or modi ed?

Click on the slider bar below to record your response. The color will change from gray to blue.

0 10 20 30 40 50 60 70 80 90 100

Currently, what percentage of ChildFN's education, services, or therapies are you now responsible for delivering (do not consider education, services, or therapies that you were responsible for delivering prior to COVID-19 changes)?

Click on the slider bar below to record your response. The color will change from gray to blue.

0 10 20 30 40 50 60 70 80 90 100

How well are you coping with the added responsibility of delivering ChildFN's education, services, or therapies?

- ◯ Completely
- ◯ Moderately
- ◯ Minimally
- ◯ Not at all

Currently, many school systems and health professionals are implementing online or remote delivery of services, therapies and medical supports (telehealth/telemedicine). Is ChildFN currently receiving any services, therapies or medical supports using this approach?

- ◯ Yes
- ◯ No

Which of the following have changed to online or remote delivery? *Check all that apply.*

- Early intervention services
- ABA services or other behavioral therapy
- Mental health services
- Medical services
- Speech and Language therapy
- Physical or Occupational therapy
- Special education services
- Other education services
- Recreational services
- Adult disability services
- Other

Please specify “other” services:

To what extent do you think ChildFN is benetting from <u>early intervention services</u> delivered online or remotely?

- SigniIcantly
- Moderately
- Minimally
- Not at all

To what extent do you think ChildFN is benetting from <u>ABA services or other behavioral therapy</u> delivered online or remotely?

- SigniIcantly
- Moderately
- Minimally
- Not at all

To what extent do you think ChildFN is benetting from <u>mental health services</u> delivered online or remotely?

- SigniIcantly
- Moderately
- Minimally
- Not at all

To what extent do you think ChildFN is benetting from <u>medical services</u> delivered online or remotely?

- Signilcantly
- Moderately
- Minimally
- Not at all

To what extent do you think ChildFN is benetting from <u>speech and language therapy</u> delivered online or remotely?

- Signilcantly
- Moderately
- Minimally
- Not at all

To what extent do you think ChildFN is benetting from <u>physical or occupational therapy</u> delivered online or remotely?

- Signilcantly
- Moderately
- Minimally
- Not at all

To what extent do you think ChildFN is benetting from <u>special education services</u> delivered online or remotely?

- Signilcantly
- Moderately
- Minimally
- Not at all

To what extent do you think ChildFN is benetting from <u>other education services</u> delivered online or remotely?

- Signilcantly
- Moderately
- Minimally
- Not at all

To what extent do you think ChildFN is benetting from <u>recreational services</u> delivered online or remotely?

- Signilcantly
- Moderately
- Minimally
- Not at all

To what extent do you think ChildFN is benetting from adult disablity services delivered online or remotely?

- SigniIcantly
- Moderately
- Minimally
- Not at all

To what extent do you think ChildFN is benetting from other services delivered online or remotely?

- SigniIcantly
- Moderately
- Minimally
- Not at all

Has ChildFN had a need to access any of the following providers since the COVID-19 changes? *Check all that apply.*

- General practitioner
- Psychiatry
- Neurology
- Gastroenterology
- Psychology
- Other subspecialties (such as endocrinology, dentistry)

Please specify "other" subspecialties:

How did ChildFN access the general practitioner services?

- Through telehealth or telemedicine
- Through at-home appointments
- Through in-person oPce appointments
- My child could not access this provider

How did ChildFN access the psychiatry services?

- Through telehealth or telemedicine
- Through at-home appointments
- Through in-person oPce appointments
- My child could not access this provider

How did ChildFN access the neurology services?

- Through telehealth or telemedicine
- Through at-home appointments
- Through in-person oPce appointments
- My child could not access this provider

How did ChildFN access the gastroenterology services?

- Through telehealth or telemedicine
- Through at-home appointments
- Through in-person oPce appointments
- My child could not access this provider

How did ChildFN access the psychology services?

- Through telehealth or telemedicine
- Through at-home appointments
- Through in-person oPce appointments
- My child could not access this provider

How did ChildFN access the other subspecialty services?

- Through telehealth or telemedicine
- Through at-home appointments
- Through in-person oPce appointments
- My child could not access this provider

Since the COVID-19 changes, which of the following have you experienced overall? *Check all that apply.*

- ChildFN's routine medical appointments have been canceled or postponed
- ChildFN's scheduled medical procedures or treatments have been canceled or postponed
- I have had diculty reaching or speaking to ChildFN's doctor(s)
- I have had trouble accessing ChildFN's medications or getting prescriptions lled
- I have had trouble managing or administering ChildFN's medications
- I have trouble a ording ChildFN's medications, treatments or therapy
- I have lost access to a clinical trial

What would help you best manage ChildFN's medications? *Check all that apply.*

- Reminders or notiUcations to administer medication
- Help with cost of medications
- Access to re lls or having enough medication at home
- Help adjusting the dose of medication
- Other

Please specify "other" help with managing medications:

To what extent do you think ChildFN is bene tting from <u>medical provider appointments</u> delivered online or remotely?

- SignIcantly
- Moderately
- Minimally
- Not at all

When services, therapies or medical supports are delivered online or remotely, what is or is not working?

NEXT

You may close this window at any time to **Save and Exit**.

Any data entered on this page will be lost. All previous pages will be saved.

SIMONS SEARCHLIGHT

Impact of COVID-19 on Services and Mental Health (Follow-up 1)

Section 2: Impact on parent employment and Rnancial stability

Which of the following options best describes your employment status in January/February 2020 (before COVID-19 changes)?

- ◯ Full-time
- ◯ Part-time
- ◯ Self-Employed
- ◯ Other
- ◯ Did not have paid work

To what extent has your employment status been negatively impacted due to COVID-19?

- ◯ SigniIcantly
- ◯ Moderately
- ◯ Minimally
- ◯ Not at all

What sorts of changes are you experiencing with regard to employment due to COVID-19?
Check all that apply.

- ☐ Working remotely all or part of the time
- ☐ Working, but with reduced hours
- ☐ Furloughed with pay (full or partial)
- ☐ Furloughed without pay
- ☐ Laid o
- ☐ High risk of being exposed to the novel coronavirus while working

- Working at home while taking care of dependent children
- Taking a full or partial leave from work in order to care for dependent children
- Other

How well are you coping with changes in your employment?

- Completely
- Moderately
- Minimally
- Not at all

How would you describe the nancial stability of your household in January/February 2020 (before COVID-19 changes)?

- Excellent
- Very good
- Good
- Fair
- Poor

To what extent has your household's nancial stability been negatively impacted by COVID-19?

- SigniIcantly
- Moderately
- Minimally
- Not at all

In 2019, what was your total annual household income?

- Less than $20,000
- $21,000 - $35,000
- $36,000 - $50,000
- $51,000 - $65,000
- $66,000 - $80,000
- $81,000 - $100,000
- $101,000 - $130,000
- $131,000 - $160,000
- Over $161,000
- Prefer not to answer

SIMONS SEARCHLIGHT

Impact of COVID-19 on Services and Mental Health (Follow-up 1)

Section 3: Impact on child, parent and family well-being

<u>During the past week</u>, how would you describe ChildFN's emotional or mental health?

- ○ Excellent
- ○ Very good
- ○ Good
- ○ Fair
- ○ Poor

Since the COVID-19 changes, have you seen an increase or worsening in your child/dependent for any of the following? *Check all that apply.*

- ☐ Repetitive behaviors
- ☐ Aggression
- ☐ Self injury
- ☐ Anxiety
- ☐ Sleep disruption
- ☐ Refusal to participate in daily activities
- ☐ Developmental regression
- ☐ Mood disturbances
- ☐ Feeding issues
- ☐ Seizures
- Other

Please specify "other" issues that have increased or worsened:

<u>During the past week</u>, how would you describe <u>your own</u> emotional or mental health?

- ○ Excellent
- ○ Very good
- ○ Good
- ○ Fair
- ○ Poor

To what extent do you feel stressed or overwhelmed by the disruption in ChildFN's services, therapies or medical supports?

- ○ Extremely
- ○ Moderately
- ○ Minimally
- ○ Not at all

Which of the following activities have you been doing to cope with COVID-19 related life changes in the <u>last month</u>? *Check all that apply.*

- ☐ Exercise or getting outdoors to enjoy nature
- ☐ Practicing meditation or mindfulness
- ☐ Listening to music
- ☐ Drinking alcohol
- ☐ Taking a recreational drug, or misusing prescription medicine
- ☐ Smoking, vaping or chewing tobacco
- ☐ Seeking counseling
- ☐ Seeking support from social network
- ☐ Pursuing hobbies or other creative activities
- ☐ Gardening or making home repairs
- ☐ Watching TV, movies, or other media
- ☐ Reading
- ☐ Spiritual activities or support
- ☐ Learning a new skill
- ☐ Eating more or less than normal
- ☐ Sleeping more or less than normal
- ☐ Spending time with members of my household
- ☐ Spending time with a pet

Other

☐ None

Please specify "other" activities:

In the past week, how often have you felt nervous, anxious, or on edge?

- ◯ Rarely or none of the time (less than 1 day)
- ◯ Some or a little of the time (1-2 days)
- ◯ Occasionally or a moderate amount of time (3-4 days)
- ◯ Most or all of the time (5-7 days)

In the past week, how often have you felt depressed?

- ◯ Rarely or none of the time (less than 1 day)
- ◯ Some or a little of the time (1-2 days)
- ◯ Occasionally or a moderate amount of time (3-4 days)
- ◯ Most or all of the time (5-7 days)

In the past week, how often have you felt lonely?

- ◯ Rarely or none of the time (less than 1 day)
- ◯ Some or a little of the time (1-2 days)
- ◯ Occasionally or a moderate amount of time (3-4 days)
- ◯ Most or all of the time (5-7 days)

In the past week, how often have you felt hopeful about the future?

- ◯ Rarely or none of the time (less than 1 day)
- ◯ Some or a little of the time (1-2 days)
- ◯ Occasionally or a moderate amount of time (3-4 days)
- ◯ Most or all of the time (5-7 days)

In the past week, how often have you had physical reactions, such as sweating, trouble breathing, nausea, or a pounding heart, when thinking about your experience (e.g., social distancing, loss of income/work, concerns about infection) with the coronavirus/COVID-19 pandemic?

- ◯ Rarely or none of the time (less than 1 day)
- ◯ Some or a little of the time (1-2 days)
- ◯ Occasionally or a moderate amount of time (3-4 days)

- Most or all of the time (5-7 days)

Overall, how concerned are you about the impact of COVID-19 on your family or household?

- Extremely
- Moderately
- Minimally
- Not at all

Please indicate where you and your family currently are right now, in terms of crisis, by selecting one of the following statements:

- 1 = Everything is ne, my family and I are not in crisis at all
- 2 = Everything is ne, but sometimes we have our diVculties
- 3 = Things are sometimes stressful, but we can deal with problems if they arise
- 4 = Things are often stressful, but we are managing to deal with problems when they arise
- 5 = Things are very stressful, but we are getting by with a lot of e ort
- 6 = We have to work extremely hard every moment of every day to avoid having a crisis
- 7 = We won't be able to handle things soon. If one more thing goes wrong, we will be in crisis
- 8 = We are currently in crisis, but are dealing with it ourselves
- 9 = We are currently in crisis, and have asked for help from crisis services (Emergency room, hospital, community crisis support)
- 10 = We are currently in crisis, and it could not get any worse

PREVIOUS NEXT

You may close this window at any time to **Save and Exit**.

Any data entered on this page will be lost. All previous pages will be saved.

SIMONS
SEARCHLIGHT

Impact of COVID-19 on Services and Mental Health (Follow-up 1)

Section 4: Other information

Since the COVID-19 changes, have you considered taking ChildFN to the hospital emergency room or urgent care clinic for any reason?

- ◯ Yes
- ◯ No

What was the reason you considered taking ChildFN to the hospital emergency room or urgent care clinic? *Check all that apply.*

- ☐ Accidental injury
- ☐ Seizures
- ☐ Psychiatric emergency
- ☐ Suspected COVID-19 symptoms
- ☐ Other condition/symptoms

When you considered taking ChildFN to the hospital emergency room or urgent care clinic, what did you decide to do? *Check all that apply.*

- ☐ Took ChildFN to emergency room or urgent care clinic, no delay
- ☐ Took ChildFN to emergency room or urgent care clinic, but delayed due to COVID-19 fears
- ☐ Called ChildFN's doctor for advice
- ☐ Scheduled telehealth/telemedicine appointment instead
- ☐ Did not go to emergency room or urgent care due to COVID-19 fears
- ☐ Did not go to emergency room or urgent care due to other reasons
- ☐ Other decision

Which of these recommendations did you follow during the past week? *Check all that apply.*

- [ ] Avoid crowded places
- [ ] Avoid public places
- [ ] Keep your distance from others (6 feet)
- [ ] Change school or work arrangements
- [ ] Quarantine yourself if you have symptoms
- [ ] None

In the past week, has anyone in your household had symptoms that were concerning for COVID-19?

- ○ Yes
- ○ No

Has anyone in your family or household tested positive for COVID-19?

- ○ Yes
- ○ No

Do you know anyone personally (outside of your family or household) who has tested positive for COVID-19?

- ○ Yes
- ○ No

Do you know anyone personally who has been hospitalized from COVID-19?

- ○ Yes
- ○ No

Do you know anyone personally who has died from COVID-19?

- ○ Yes
- ○ No

PREVIOUS | NEXT

You may close this window at any time to **Save and Exit**.

Any data entered on this page will be lost. All previous pages will be saved.

Impact of COVID-19 on Services and Mental Health (Follow-up 1)

Section 5: Additional Comments

During the past month, what have been the biggest challenges for ChildFN and for your family?

During the past month, what have been the biggest successes for ChildFN and for your family?

PREVIOUS SUBMIT

You may close this window at any time to **Save and Exit**.

Any data entered on this page will be lost. All previous pages will be saved.
